# Non-goal-driven eye movement after visual search task

**DOI:** 10.16910/jemr.15.2.2

**Published:** 2022-06-02

**Authors:** Ayumi Takemoto, Atsushi Nakazawa, Takatsune Kumada

**Affiliations:** OMRON Corporation, Kyoto, Japan; University of Latvia, Riga, Latvia; Kyoto University, Kyoto, Japan

**Keywords:** eye movement, saccades, attention, gaze, scan path

## Abstract

We investigated the functions and mechanisms of non-goal-driven eye movements, which are defined as eye movements induced when looking at visual stimuli on a display without engaging in a specific task or looking at a display without any visual stimuli or tasks. In our experiment, participants were asked to perform a visual search task on a display, which was followed by a rest period in which stimuli remained on the display or all stimuli were erased. During the rest period, the participants were asked to only look at the displays without engaging in any visual or cognitive tasks. We mainly analyzed the gaze-shift patterns in both task and rest periods, in which eye movements were classified in accordance with the angles of saccade directions in two consecutive saccades. The results indicate a significant difference between goal-driven eye movements, which were observed in the task period, and nongoal- driven eye movements, which were observed in the rest period. Scanning gaze-shift patterns dominated the task period, and backward and corrective-saccade-like gaze-shift patterns dominated the rest period. The gaze-shift pattern was affected by the task-difficulty during the task period. From these findings, we propose a model describing the oculomotor system in terms of goal-driven and non-goal-driven eye movements. In this model, the engagement levels of top-down and bottom-up control change along with task difficulty and are affected by the gaze-shift patterns during a visual search task. Decoupling of top-down control from the oculomotor system during a rest period induces backward saccades, resulting in fixation around the central part of a display. Therefore, we suggest that non-goaldriven eye movements play a crucial role in maintaining the readiness of the oculomotor system for the next search task.

## Introduction

Our eyes move most of the time we are awake. When we search for a pen on a table or for a cafe on an unfamiliar street, our eyes move on purpose. The cognitive mechanisms of such goal-driven movements have been the subject of many studies. However, eyes sometimes move without any purpose, but the cognitive mechanisms and functions of such non-goal-driven eye movements have not been extensively investigated. We investigated the cognitive mechanisms of non-goal-driven eye movements that are affected by the aftereffects of preceding goal-driven eye movements.

In most behavioral studies, eye movements are measured under the experimental conditions that some tasks or goals are explicitly given to participants. Under such a condition, almost all studies agreed that eye movements are controlled by a "top-down" process, which is driven by the goal of a task, and a "bottom-up" process, which is invoked by incoming visual stimuli (7; 12; 15). When a goal of eye behavior is given, a top-down control to the goal is formed, and eye movements repeat until the goal is achieved (23; 34). However, under such top-down control, the bottom-up stimulus-driven control is effective to guide saccades when visually salient objects are present in a visual scene. Although it has been reported that eyes can become focused on a salient object even when participants are given a clear goal of saccade (28), both controls work collaboratively in most daily situations.

The mechanisms of non-goal-driven eye movements are less known compared with those of goal-driven eye movements or attentional control. Only a few studies investigated eye movements, possibly spontaneous, during non-visual tasks (3; 9), which are referred to as non-visual gaze patterns (NVGPs). NVGPs are more frequently induced by long-term memory search tasks, such as verbal-fluency tasks, than by working-memory tasks (18). It has been suggested that the process of scanning for information in long-term memory interacts with the brain mechanisms, including the superior colliculus, controlling eye movements.

We conducted an experiment to investigate non-goal-driven eye movements immediately after goal-driven eye movements. We presented stimuli on a display during a visual task then presented the same stimuli or a blank display without giving any goal of eye movements during a rest period. To assess the impact of the preceding visual task on eye movements on the following after-task phases, we manipulated the task difficulty of the visual search. If the effect of visual tasks on the mechanisms of eye movements persisted into the after-task phases, we expected that eye movement in these phases should be affected by the task difficulty in the preceding visual task.

We used a visual search task for three reasons. First, the task requires frequent eye movements to achieve task goals. Second, task-difficulty can be easily manipulated by changing the feature relationship between a target and distractor (8; 33). Third, the eye-movement mechanisms have been extensively examined not only on the human-behavior level (10; 36) but also on the physiological level of animal or human brains in visual tasks (5; 19; 20). The contribution of top-down and bottom-up control can be manipulated by the target-distractor relationship. When a target is defined by the difference in visual features, eye movements tend to be guided by bottom-up control. When a target is defined by a combination of visual features, a serial, item-by-item, search is required under top-down control.

We also assumed that a person's mental state, particularly on- or off-task, affects eye movements in the after-task phases. In one study involving a reading task, eye movements became slower or less frequent during a period prior to a participant's report of mind-wandering compared with a period prior to reporting of being on-task (concentrating) (31). Another study reported that the saccade frequency was lower and the fixation dispersion was more limited in mind-wandering states than in attentive states during video lectures (13). Therefore, compared with the task phase, people are likely to be in a mind-wandering state in the after-task phase because no specific goal is given.

In previous eye-movement studies, basic metrics of eye movements, i.e., saccade frequency, pupil size, blink ratio, and amplitude, were mainly analyzed (14; 21; 24; 29). In addition to these basic metrics, to contrast goal-driven to non-goal-driven eye movements, we conducted two analyses. We first analyzed the spatial distribution of fixations. It is plausible that fixations would distribute widely in the task phase, relative to the after-task phases, because a target could present in any position on a display. There was no need to move the eyes around the display in the after-task phases. Therefore, fixation should be strongly biased to the center of a display in after-task phases. We then analyzed consecutive fixations, specifically focusing on the relationship of the amplitudes on two successive saccades and the angle of direction change of these saccades. This analysis, referred to as a gaze-shift-pattern analysis, enables us to examine the occurrence of specific eye movements. For example, corrective saccades, in which a relatively large saccade is accompanied by a small saccade (1; 2; 4), have been observed for fixating a target in visual search tasks.

We focused on non-goal-driven eye movements during the inter-trial intervals of a visual search task. In our experiment, participants were asked to perform a visual search task (either easy or difficult), which was followed by a 10-sec rest period during which either the stimuli remained on a display or were eliminated from the display. The stimuli remaining on the display were used to assess the effect of a stimulus-driven factor on eye movements by comparing the eye movement when no stimuli were displayed in an after-task phase. Following the after-task phases, participants reported if they focused on the task (on-task) or not (off-task).

We focused on three points: (1) the differences between goal-driven and non-goal-driven eye movements, (2) aftereffects of the task difficulty of the preceding task on non-goal-driven eye movements, and (3) effect of mental state, i.e., on-task or off-task, on non-goal-driven eye movements. We introduced two novel features to grasp the gaze behaviors: (1) gaze distributions that represent the distribution of fixations in a display and (2) gaze-shift pattern that represents the movements of continuous fixations.

## Methods

### Participants

Fifteen healthy participants were recruited from Kyoto University. All participants signed an informed consent before the experiment and received 1,500 JPY (approximately 15 USD) for participating. The data from four participants were excluded from the analysis because one participant did not understand the rules of the experiment, a camera issue occurred during one participant's experimental session, and two participants slept during most of the experiment and missed more than 10% of their self-report events. Therefore, the data reported here were collected from 11 participants (7 men, 4 women, age range 19-25, mean 22.4, standard deviation (SD) = 1.37).

### Apparatus

The stimuli presentation and response collection were controlled using PsychoPy (22), a software library written in Python for Intel-based PCs. The stimuli were presented on a monitor (Acer KA270H, 1920 x 1080 pixels, 59.7 x 33.6 cm) at a viewing distance of 60 cm. The participants used a button (Kokuyo, ELA-FP1) for responding to either finding a target during the task phase or reporting being on-/off-task following the after-task phases. Their eye movements were monitored using an eye-tracker (Tobii 4C) with a 90-Hz refresh rate, by using the Tobii Python SDK. The tracker was calibrated before every session.

### Procedure

**Figure 01.**
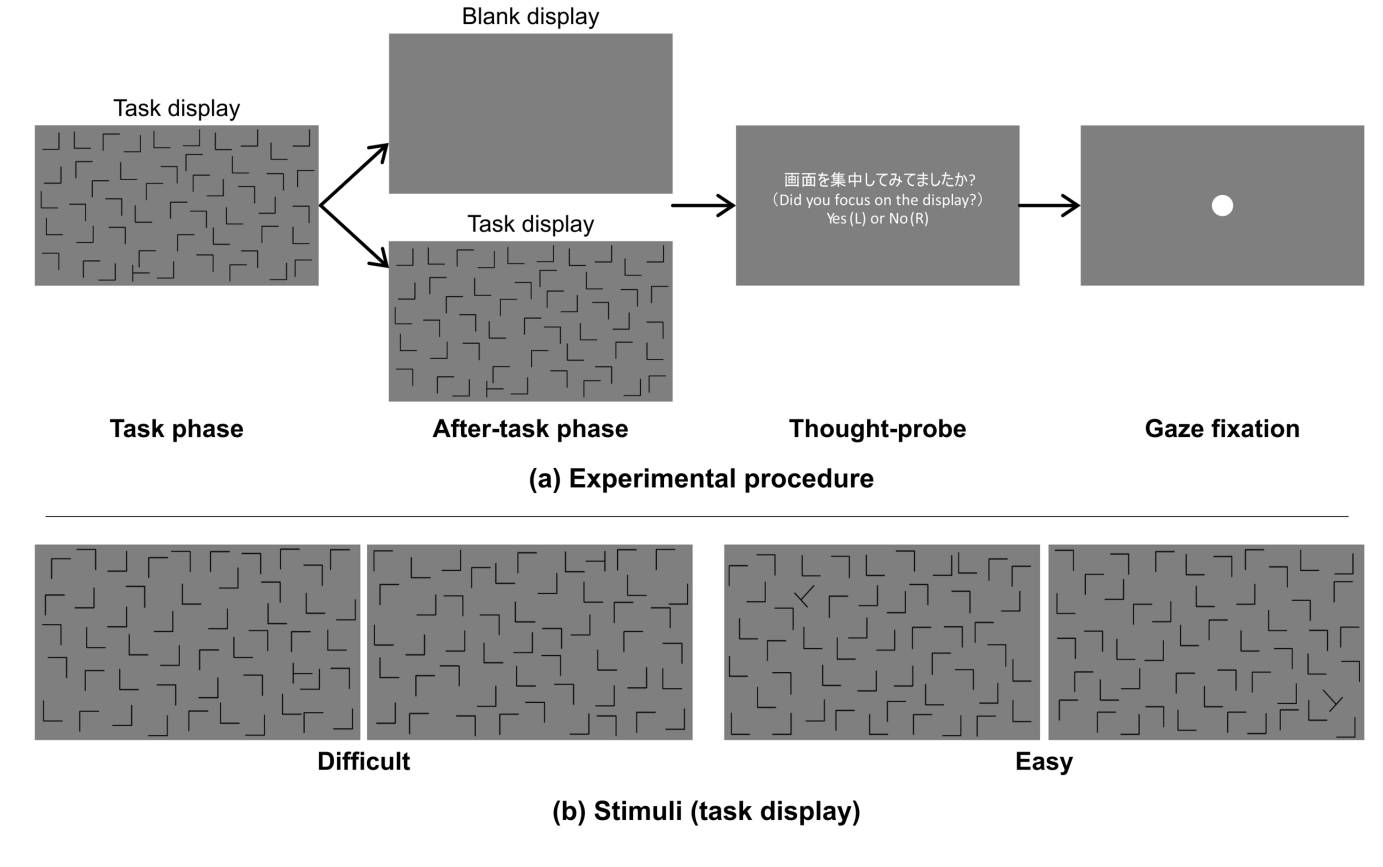
(a) Time schedule of stimulus presentation and (b) examples of visual search displays

An experimental session consisted of 200 trials (only one participant performed 240 trials) and lasted approximately 50 min. Each trial consisted of a task phase and after-task phases as shown in Figure 01(a). In the task phase, a visual search display was presented for 60 sec or until the participant responded. The participants were required to search for a target and press a button immediately when they found the target. The duration between the presentation of a search display and button press was recorded as the reaction time (RT). After the task phase, the trial proceeded to an after-task phase, in which either a blank (after-task(blank)) or task display (after-task(stimulus)) was shown. The blank display was a uniform gray, while the task display was the same as the search display used in the task phase. The blank or search display was randomly selected with a probability of 50% for each. The after-task phases lasted 10 sec, and the participants were asked to avoid head movements for that duration. Afterward, a thought-probing display was presented, in which the participants were asked to report if they were on- or off-task at the last seconds by pressing one of two buttons; specifically, pressing the left button if they were on-task or the right one if off-task, which is a common methodology to ask participants' on- or off-task states (32). However, we did not report the data in this study because some participants’ reports polarized to one state, thus were not able to compare the effect of the status within participant. Finally, a fixation display was presented until the participant pressed a button (fixation phase). The experiment was conducted in accordance with a protocol approved by the ethical committee of Omron Co. [OMRON: RD-ECE-00010].

### Materials

Figure 01(b) shows examples of the search display. We used "**L**" shapes as distractors and a "**T**" shape as the target. The shapes were black and were each approximately 3.5 x 3.5 cm (3.34 x 3.34 deg.) in height and width on the monitor. We used two different task-difficulty levels of visual search, difficult and easy. The target was rotated ± 90 deg in the difficult-task search display but ± 45 or ± 135 deg. in the easy-task search display. Both types of displays consisted of 49 distractors and 1 target. All shapes were located in the central rectangular area (59.7 x 33.6 cm) of the monitor.

### Analysis

1. Eye movement analysis

From the recorded eye positions of the participants' left eyes, three types of data were obtained: All data were analyzed using MATLAB (MathWorks, Natick, MA) and Python. We used the eye position data from all Task and after-task phases in which the participants had their eyes opened for more than 80% of the frames. We then detected the fixation points ${𝐱}_{𝐤}\in R^{2}$ (k=1,…,K: the number of the fixation) by using the EyeMMV toolbox (16); we assumed fixation occurred where several contiguous gaze points were all located within a degree radius.

2. Saccade frequency

The saccade frequency in every phase was used as a metric. Specifically, we computed the number of saccades occurring in a second and used the value as the saccade frequency. In the task phase, because the saccade frequency varied depending on the RT, the saccade frequency was given by (the number of saccades) x 1/RT. In the after-task phases, we simply counted the number of saccades then computed (the number of saccades) x 1/10 because the duration was 10 sec.

3. Gaze-shift pattern

**Figure 02.**
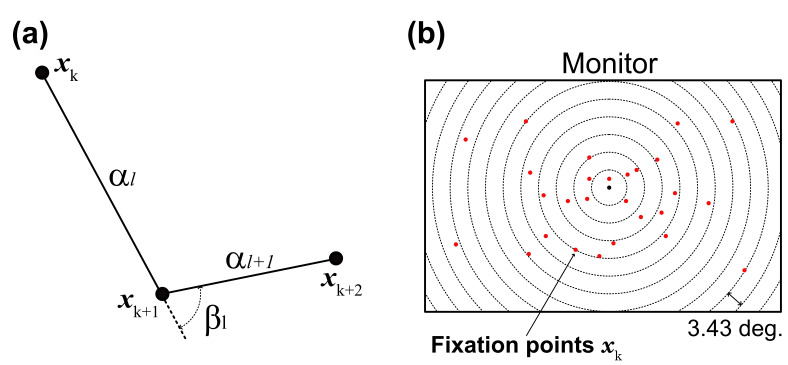
Eye-position analysis. (a) Gaze-shift angle ( $\beta_{l}$) was computed from three adjacent fixation points ( ${𝐱}_{𝐤-1},{𝐱}_{𝐤},{𝐱}_{𝐤+1}$) and corresponding saccade amplitudes ( $\alpha_{k-1},\alpha_{k}$). These parameters were used for clustering gaze-shift patterns. (b) Gaze distribution was obtained by density of gaze fixations in each series of concentric circles. Radii of circles were set to 1/20 diagonal of the monitor (3.43 deg.) x N deg. (N = 1,…,20).

We developed a gaze-shift-pattern analysis that encodes three consecutive fixations. Past studies (6; 11) reported that a saccade in a sequence affected the following ones, and the effect may change according to the task given to participants. Thus, in this study, we investigated whether eye-movement sequences were affected by phases and task difficulty, namely, in which direction the gaze moved, similar or opposite direction**.** Given three consecutive fixations $x_{k},\;x_{k+1},\;{and,\;\;x}_{k+2}$, their angular displacements (saccade amplitudes) $\alpha_{k}\;\text{ and }\;\alpha_{k+1}$ and their angle $\beta_{l}$ were computed as illustrated in Figure 02(a). Thus, we obtained K-2 three-dimensional feature vectors $f_{l}$ = [ $\alpha_{l}$, $\alpha_{l+1}$, $\beta_{l}$], where l=1,…,K-2 from $x_{k}$. We then classified the gaze positions into five patterns on the basis of $\beta_{l}$, with unique labels assigned to each pattern: pattern 1 = $\left\{\beta_{l}|0\leq \beta_{l}<36\right\}$, pattern 2 = $\left\{\beta_{l}|36\leq \beta_{l}<72\right\}$, pattern 3 = $\left\{\beta_{l}|72\leq \beta_{l}<108\right\}$, pattern 4 = $\left\{\beta_{l}|108\leq \beta_{l}<144\right\}$, and pattern 5 = $\left\{\beta_{l}|144\leq \beta_{l}<180\right\}$.

4. Gaze distribution

Figure 02(b) illustrates the idea we used in computing gaze distribution. Specifically, we defined occupancy areas consisting of concentric circles the centers of which were the center of the monitor, and counted the number of fixation points ${𝐱}_{𝐤}$ in each area. The radii of the circles were set to 1/20 diagonal of the monitor (3.43 deg.) x N (N = 1,…,20). Finally, we normalized the number of fixation points by the occupancy area and used the result as a 11-dimensional gaze distribution metric.

## Results

### Accuracy and mean reaction time

The accuracy of visual search tasks was very high in both difficult (mean(M)= 99.00%, SD=1.26%) and easy (M=99.12%, SD=1.01%) tasks, and there was no significant difference between them (t(10) = 0.38, p = .710), indicating that there was no speed-accuracy tradeoff between these two conditions. The RTs in the difficult task (M=2.39 sec, SD=0.72) were approximately twice those in the easy task (M=1.07 sec, SD=0.27), with t(10) = -8.57, p < .01. This indicates that it took longer to find the target in the difficult task than in the easy task.

### Gaze features as function of phase and task difficulty

**Figure 03.**
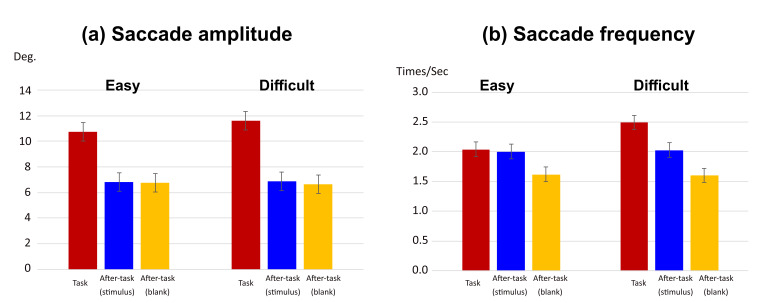
Effect of visual task difficulty on saccades. (a) saccade amplitude and (b) saccade frequency in task phase. Error bars in (a) and (b) indicate 95% confidential interval based on Loftus, G. R. and Masson, M. E. (17) procedure.

The saccade amplitude and frequency are shown in Figure 03. We conducted a two-way analysis of variance (ANOVA) with phase and task difficulty as the main factors. In this and subsequent ANOVAs of this study, a Huynh–Feldt correction was applied when the assumption of sphericity was not met by the Mendoza test. A Shaffer's modified sequentially rejective Bonferroni procedure was also used for correcting multiple comparisons.

For the saccade amplitude (see Figure 03(a)), the main effects of phase and task difficulty level were significant (F(1.59,15.86) = 45.97, p < .0001, $\eta^{2}$= .538; F(1,10) = 5.99, p = .034, $\eta^{2}$ = .002, respectively). Interaction between phase and task difficulty was also significant (F(1.25,12.46) = 4.77, p = .042, $\eta^{2}$ = .006). A simple main-effect analysis of the interaction showed that there was a significant simple main effect of the task difficulty only in the task phase (F(1,10)=9.82, p = .0106, $\eta^{2}$ = 0.106). The amplitude was larger in the difficult task than the easy task. The simple main effect of the phase was significant in both task difficulty level (easy task, F(1.42,14.23)=26.54, p <.0001, $\eta^{2}$ =.478; difficult task, F(1.82,18.21)=67.03, p <.0001, $\eta^{2}$=.601). For both task difficulty levels, the amplitude in the task phase was larger than that in the after-task(stimulus) and the after-task(blank) phases, and there was no difference between the two after-task phases.

Similarly, for the saccade frequency (see Figure 03(b)), the main effects of phase and task difficulty were significant (F(1.88, 18.77) = 26.46, p < .0001, $\eta^{2}$ = .492 and F(1,10) = 78.98, p < .0001, $\eta^{2}$ = .039, respectively). Interaction between phase and task difficulty was also significant (F(1.82,19.15) = 52.80, p < .0001, $\eta^{2}$ = .077). A simple main-effect analysis of the interaction showed that there was a significant simple main effect of task difficulty only in the task phase (F(1,10) = 97.81, p < .0001, $\eta^{2}$ = 0.524). Saccade frequency was higher in the difficult task than in the easy task. The simple main effect of phase was also significant for both task-difficulty levels (easy task, F(2,20) = 13.84, p = .0002, $\eta^{2}$ = .381; difficult task, F(1.64,16.37) = 39.72, p < .0001, $\eta^{2}$ = .697). In the easy task, although there was no difference between the task and after-task(stimulus) phases, they were significantly larger than the after-task(blank) phase. In the difficult task, he frequency in the task phase was higher than that in the after-task(stimulus) and the after-task(blank) phases, and that in the after-task(stimulus) phase was higher than that in the after-task(blank) phase.

### Gaze shift angle

**Figure 04.**
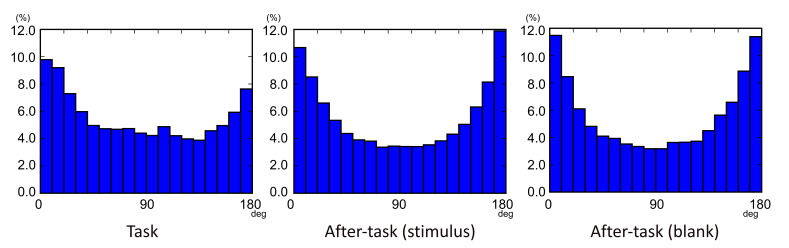
Gaze-metric statistics: distributions of gaze-shift angles ( βs).

Eye-movement sequences in three consecutive saccades were examined in each task phase. Figure 04 shows histograms of the gaze-shift angle β in consecutive saccades. The βs were generally biased around 0 and 180 deg., which indicates that gaze shifts tended to occur frequently in the opposite (180 deg.) and straight ahead (0 deg.) directions, relative to other directions. However, these trends were somewhat different among phases. Specifically, the βs between 0 and 60 deg. were more frequent than other angles in the task phase, whereas the distribution of angles was almost symmetric in the after-task phases.

### Spatial distribution of fixation position

**Figure 05.**
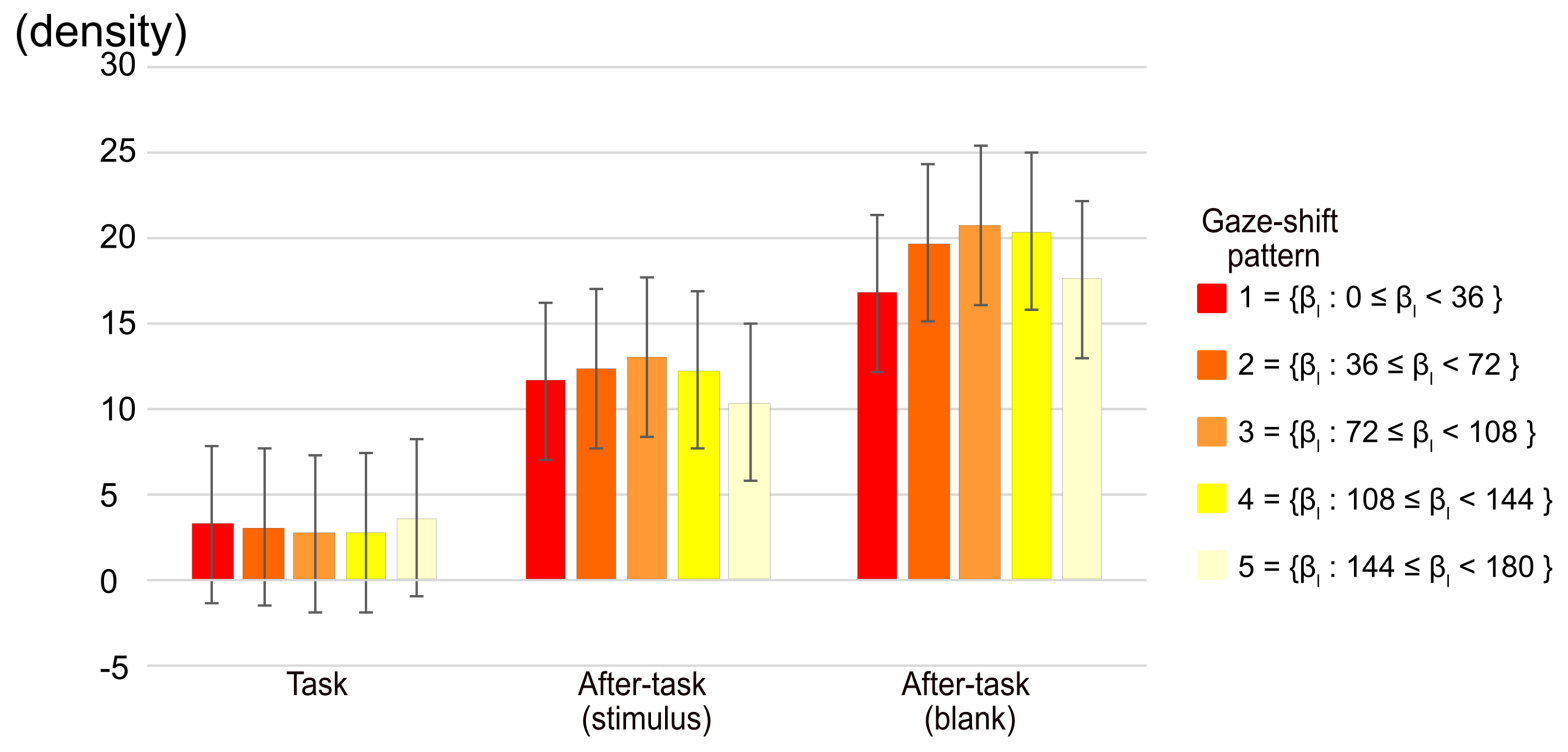
Gaze density of area within 1/20 diagonal of monitor from central point of display. Error bars indicate 95% confidential interval based on Loftus, G. R. and Masson, M. E. (17) procedure.

We calculated the distribution of fixation positions as a function of the radius from the center of a display. We then examined the distribution in terms of the five gaze-shift patterns, categorized on the basis of β, as shown in Figure 05. The number of fixations located within a radius of 3.43 deg. from the center of the display were subjected to a two-way ANOVA with phase and gaze-shift pattern as main factors. The main effect of the phase and the phase x gaze-shift pattern interaction were significant (F(2,20) = 9.30, p = .0014, $\eta^{2}$ = .296 and F(6.85,68.45) = 2.35 p = .0336, $\eta^{2}$ = .005, respectively). The multiple comparison among phases showed that there were significant differences between the task and both after-task phases, indicating that the fixation on the central region was more in the after-task phases than in the task phase. The simple main-effect analysis of the interaction shows that the effect of gaze-shift pattern was significant only in the after-task(blank) phase (F(3.7,37.02) = 2.76, p = .0450, $\eta^{2}$ = .012), although multiple comparisons among phases did not show a significant difference among any comparisons of patterns. As shown in Figure 05, however, the fixation to the center tended to be smaller in patterns 1 and 5.

### Gaze-shift patterns regarding phase and task difficulty

**Figure 06.**
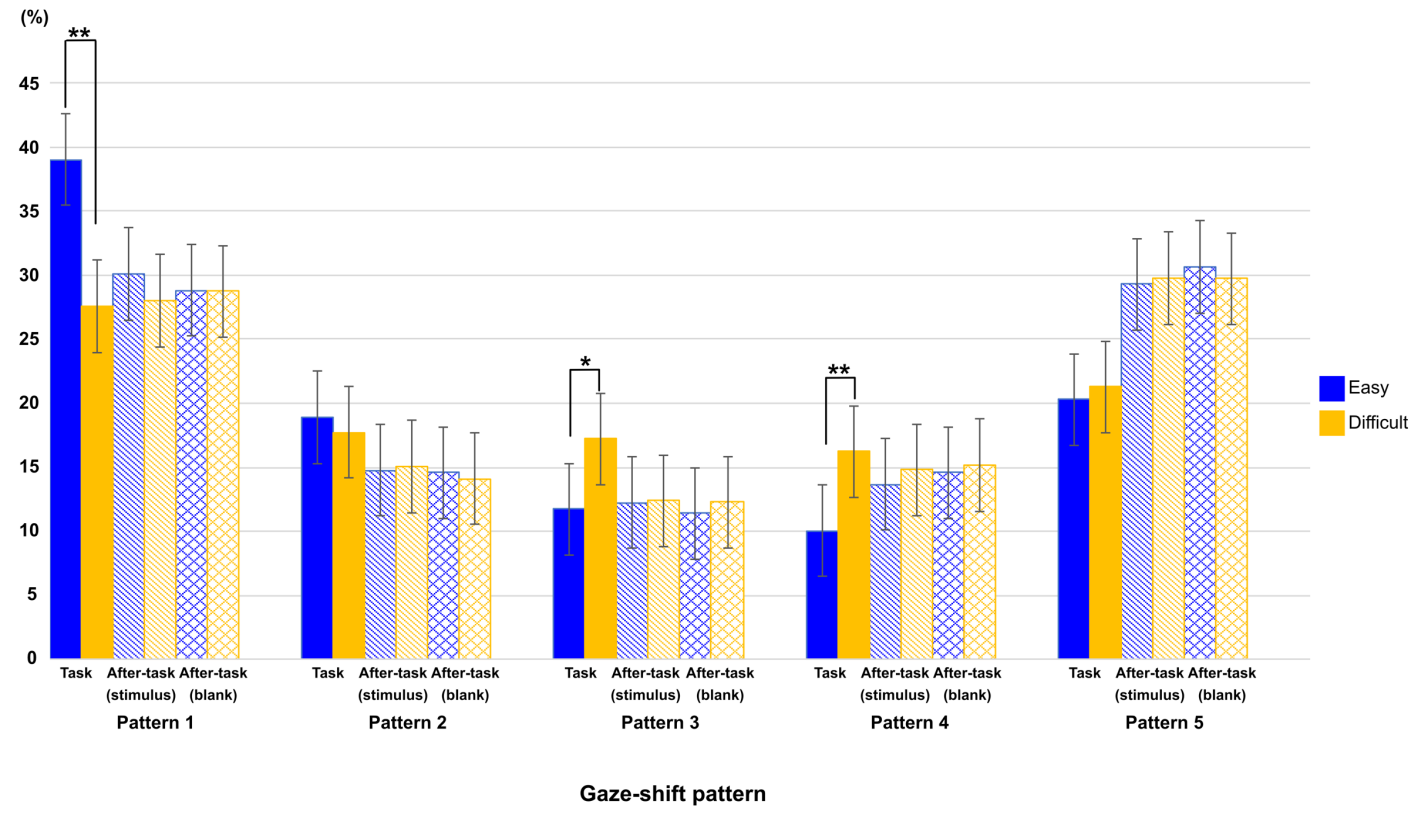
Ratio of each type of eye-shift pattern in each phase for both task-difficulty levels. Error bars indicate 95% confidential interval based on Loftus, G. R. and Masson, M. E. (17) procedure. ** : p < 0.01, *: p < 0.05

Figure 06 shows the ratio of each gaze-shift pattern between task difficulties in all phases. To show the effect of task difficulty and phase on this ratio, we conducted a two-way ANOVA with these factors as main terms, separately for each gaze-shift pattern, then the p-value by using the Bonferroni method for multiple comparison.

Interaction among task phase, gaze-shift pattern, and task difficulty was significant (F(7.36,73.56) = 12.38, p < .0001, $\eta^{2}$ = .020), and interactions between task phase and gaze-shift pattern, and between gaze-shift pattern and task difficulty was also showed significant (F(3.13,31.33) = 6.64, p = .001, $\eta^{2}$ = .060; F(4,39.98) = 16.81, p < .0001, $\eta^{2}$ = .018, respectively).

We then conducted a more detailed analysis to investigate the trend in each pattern. The results of patterns 1, 3, and 4 showed the effect of task difficulty. There were significant main effects of task difficulty (pattern 1, F(1,10) = 34.91, p < .0001, $\eta^{2}$ = .057; pattern 3, F(1,10) = 37.07, p < .0001 $\eta^{2}$ = 0.092 and pattern 4, F(1,10) = 56.21, p = .0001 $\eta^{2}$ = .1377) and interaction between task difficulty and task phase (pattern 1, F(2,20) = 34.00, p < .0001 $\eta^{2}$ = .069; pattern3, F(1.75,17.49) = 11.10, p = .0197, $\eta^{2}$ = .109 and pattern 4, F(1.49,14.91) = 14.33, p = .0100, $\eta^{2}$ = .126). A simple main-effect analysis of the interaction showed similar results in patterns 1 and 4. The main effect of phase was significant only in the easy task (pattern 1, F(1.36,13.60) = 10.37, p = .0334, $\eta^{2}$ = .187 and pattern 4, F(1.40,14.03) = 8.91, p = .0491, $\eta^{2}$ = .308). However, the main effect of phase was significant only in the difficult task in pattern 3 (F(2,20) = 11.79, p = 0.0020, $\eta^{2}$ = .364). As shown in Figure 06, multiple comparisons showed that the ratio of the task phase was larger in pattern 1 and smaller in pattern 4 than both after-task phases, only in the easy-task. In addition, the ratio of the task phase was larger than that of both after-task phases in pattern 3 only in the difficult task. In patterns 2 and 5, only the main effect of phase was significant (pattern 2, F(2,20) = 16.11, p = .0003, $\eta^{2}$ = .286 and pattern 5, F(2,20) = 10.71, p = .0034, $\eta^{2}$ = .228). Multiple comparisons showed that the ratio of the task phase was larger in pattern 2 and smaller in pattern 5 than both after-task phases. This is consistent with the results shown in Figure 04: there were larger ratios around 45 deg. and smaller ratios around 180 deg. in the task phase, relative to both after-task phases. Finally, as shown in Figure 06, all analyses showed that there was no significant difference in the ratio between difficult and easy tasks in both after-task phases.

**Figure 07.**
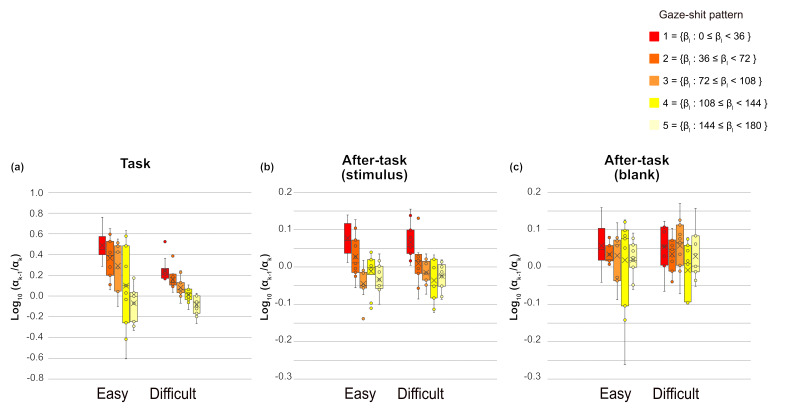
$\frac{\alpha_{k-1}}{\alpha_{k}}$ of each gaze-shift pattern and task phase for each task difficulty level. Circles are outliers, Xs are mean, lines in side boxes are median line, lines in upper boxes indicate maximum, and those in lower boxes indicate minimum. Boxes range from 25 to 75%.

We assessed $\frac{\alpha_{k-1}}{\alpha_{k}}$ which is the ratio of successive saccade amplitude, to measure the saccade sequences, then conducted a three-way ANOVA with phase, gaze-shift pattern, and task difficulty as main factors, separately for $\frac{\alpha_{k-1}}{\alpha_{k}}$ and fixation duration of $x_{k}$. For $\frac{\alpha_{k-1}}{\alpha_{k}}$, all main effects were significant (phase, F(1.13,11.33) = 23.94, p = .0003, $\eta^{2}$ = .162; task difficulty, F(1,10) = 14.56, p = .0034, $\eta^{2}$ = .023 and gaze-shift pattern, F(2.60,26.01) = 20.82, p < .0001, $\eta^{2}$ = .158). Interactions between phase and task difficulty and between phase and gaze-shift pattern were significant (F(1.03,10.26) = 13.33, p = .0041, $\eta^{2}$ =.045, F(4.82,48.2) = 14.38, p < .0001, $\eta^{2}$ =.142, respectively). Interaction between task difficulty and gaze-shift pattern and the three-way interaction were not significant. The simple main-effect analysis of interaction between phase and task difficulty showed significant main effects of phase in both easy and difficult tasks (F(1.06, 10.63) = 20.84, p = .0008, $\eta^{2}$ =.235; F(1.38,13.79) = 17.23, p =.0005, $\eta^{2}$ = .109, respectively). Multiple comparisons revealed that $\frac{\alpha_{k-1}}{\alpha_{k}}$, was smaller in the task phase than in both after-task phases and was smaller in the after-task(stimulus) phase than in the after-task(blank) phase for both task difficulty levels. In addition, the main effect of task difficulty was significant only in the task phase (F(1,10) = 14.01, p = .0038, $\eta^{2}$ = .091). The ratio was larger in the easy than in the difficult task in the task phase. The simple main-effect analysis of the phase x gaze-shift pattern interaction showed that the effects of gaze-shift pattern were significant in the task and after-task(stimulus) phases (F(2.84,28.35) = 21.29, p < .0001, $\eta^{2}$ = .376; F(4,40) = 13.89, p < .0001, $\eta^{2}$ = .429, respectively). As shown in Figure 07, the ratio tended to decrease as a function of the gaze-shift angle, shown as at pattern, in the task phase. This trend was also evident, but relatively weaker, in the after-task(stimulus) phase than in the task phase.

For fixation duration, the main effects of phase and gaze-shift pattern were significant (F(1.29,12.94) = 37.7, p < .0001, $\eta^{2}$ = .569 and F(2.33,23.31) = 8.35, p = .0012, $\eta^{2}$ = .022, respectively). Interaction between task difficulty and gaze-shift pattern was also significant (F(3.69,36.89) = 3.50, p = .0184, $\eta^{2}$ = .002). The main effects of task difficulty and other interactions were not significant. The duration in the after-task(blank) phase was significantly longer than that in other phases, and that in the after-task(stimulus) phase was longer than that in the task phase (207.4 ms in the task phase, 316.2 ms in the after-task(stimulus) phase, and 409 ms in the after-task(blank) phase). A simple main-effect analysis for interaction between task difficulty and gaze-shift pattern showed that the effect of gaze-shift pattern was significant in both task-difficulty levels (easy task, F(3.11,31.07) = 10.41, p = .0001, $\eta^{2}$ = .033; difficult task, F(2.60,25.98) = 4.12, p = .0198, $\eta^{2}$ = .013). Multiple comparisons showed that the duration of pattern 1 was shorter than other patterns, and that of pattern 2 was shorter than that of pattern 4 in the easy task. However, there was a significant difference only between patterns 1 and 3 in the difficult task

## Discussion

We examined eye movements in visual search displays and in the following displays in which participants were allowed to freely view two types of displays: the same visual search display as presented in the preceding search task (after-task(stimulus)) or without either targets or distractors (after-task(blank)). We found differences between goal-driven and non-goal-driven eye movements. Goal-driven eye movements are engaged by both top-down/goal-driven and bottom-up/stimulus-driven control, but we argue that top-down/goal-driven control is the more influential than bottom-up/stimulus-driven control. Thus, the task-difficulty level of a visual task changes the gaze-shift patterns, frequency, and amplitude of saccades. Specifically, less top-down/goal-driven control induces corrective saccades, lower frequency, and smaller amplitude, whereas more top-down/goal-driven control induces scanning or searching saccades, higher frequency, and higher amplitude. Non-goal-driven eye movement, induced during an inter-task interval, mainly consists of directly backward and corrective-like saccades, with the eye movements tending to fixate on the central part of the display to enable the oculomotor system (OMS) to rest and be ready for the next search task. In this section, we discuss the results with respect to three points presented in the Introduction then propose a model for understanding the results.

### Differences between goal-driven and non-goal-driven eye movements

First, the comparison of eye movements among phases revealed that saccade amplitude modulated depending on the task demand. Specifically, the saccade amplitude was significantly larger in the task phase than in the after-task phases. This was consistent with a previous study reporting that the saccade amplitudes were greater in a search task than in free-viewing tasks (27). Similarly, the saccade frequency decreased in the after-task(stimulus) and after-task(blank) phases relative to the task phase. The difference in saccade amplitude and frequency between the task phase and both after-task phases is due to involvement of the top-down/goal-driven control. Eye movements in the task phase were driven by top-down/goal-driven and bottom-up/stimulus-driven controls, whereas the top-down/goal-driven control might less affect in the after-task phases because the participants were not required to search for a target. However, the saccade frequency and amplitude showed different results in the after-task(blank) phase. Although there was no difference in saccade amplitude between after-task phases, saccade frequency was higher in the after-task(stimulus) phase than in the after-task(blank) phase. In the after-task(blank) phase, unlike the after-task(stimulus) phase, bottom-up/stimulus-driven control might engage less with gaze pattern because there were no items in the display. This indicates that the saccade amplitude is only affected by top-down/goal-driven control. The saccade frequency, however, is affected by both top-down/goal-driven and bottom-up/stimulus-driven controls. In other words, presentation of visual items might induce eye movements, even when no specific task-goal is given.

Second, there were several differences in eye-movement patterns between the task phase and after-task phases. As shown in Figure 04, the distribution of β differed among the phases. In all phases, a relatively large number of βs is distributed in the 0-10 and 170-180 deg. ranges. The 0-10 deg. of β indicates that two consecutive saccades occur in the similar direction. This is likely due to "corrective saccades". A corrective saccade reportedly occurs when two saccades are required to reach a target: first, a large saccade moves about 90% of the distance to the target then a smaller saccade brings the eye to the target (4). We thus argue that gaze-shift pattern 1, especially in the task phase, had the same features as a corrective saccade in which $\alpha_{k-1}$ was greater than $\alpha_{k}$. Interestingly, in the after-task phases, pattern 1 occurred as the same ratio as in the task phase. Although the ratio of saccade amplitudes in pattern 1 was smaller in the after-task phases than that in the task phase, the effect of the phase was significant in the after-task(stimulus) phase, indicating the trend in decreasing the ratio as a function of β in the phase. This suggests that "corrective saccade-like" eye movements would occur even when no task demand for searching a target was given in the after-task(stimulus) phase. As shown in Figure 07, the range of the ratio in the after-task(blank) phase was similar to that in the after-task(stimulus) phase. This suggests that "corrective saccade-like" eye movements might occur even when no visual items were present in a display. To the best of our knowledge, there was no corrective saccade-like eye movements without any visual targets. “Corrective” means saccades toward a visible target when the first saccade is incorrect. Therefore, the terminology is still controversial. However, one study reported that participants make corrective saccades even in a dark room (4). In that study, participants in a dark room looked at two very dim light spots with a visual angle of 40 deg., then the lights were extinguished. When they asked to make saccade to the location where light spots were presented, they made corrective saccades even though there were no targets in the dark. This result and our results indicate that a visual target would be sufficient for corrective saccade-like eye movements. However, we acknowledge that it is speculative.

Pattern 5 was induced mainly in the after-task phases. In pattern 5, $\beta_{k}$ was larger than 145 deg. and the $\frac{\alpha_{k-1}}{\alpha_{k}}$ ratio was the smallest among all patterns in all phases (Figure 07). This shows that, the first saccade could have gone anywhere on the display, while the second saccade moved back to the position of the first saccade, because the amplitude of the first ( $\alpha_{k-1}$) was as large as that of the second ( $\alpha_{k}$), and the gaze-shift angle ( $\beta_{k}$) was approximately 180 deg. Importantly, this "backward saccade" occurred more in the after-task phases.

To the best of our knowledge, there has been no study of backward saccades in visual tasks without top-down/goal-driven regulation. We also suggest that pattern 5 was for inducing central fixation bias during the free viewing in the after-task phases. Past studies showed that gaze fixation is biased toward the center of natural scene stimuli (i.e., centering bias) (25; 26; 30). We suggest that the central area at the monitor was the straight in front of participants’ face and would be convenient location for them. Our suggestion is consistent with Tatler et al., who reported that the center of the display is a convenient location and the eye moves close to the center of the display with the first saccade when a scene appears, even during a visual task (26).

Finally, the eye was induced more strongly to fixate on the center of the display in the after-task phases than in the task phase. When top-down/goal-driven control becomes less effective, we suggest that the eye moves toward a convenient location, likely the central part of a display. The trend in fixing on the central parts of a display increased in the after-task phases, relative to the task phase. It is plausible that the central fixation bias was attenuated because the eye moved around the display to search for a target in the task phase. In other words, both top-down/goal-driven and bottom-up/stimuli-driven controls inhibited fixation on the center of the display (i.e., centering-bias) and promoted saccades to find a target in the task phase. Furthermore, the effect of the stimuli-driven control of eye movements was found in very limited aspects of the data. As we mentioned, the saccade frequency was less in the after-task(blank) phase than in the after-task(stimulus) phase. In addition, as shown in Figure 07, the $\frac{\alpha_{k-1}}{\alpha_{k}}$ ratio tended to depend on patterns in the after-task(stimulus) but not in the after-task(blank) phase. The central-fixation bias was stronger in the after-task(stimulus) phase than in the after-task(blank) phase (Figure 05). These results suggest that visual stimuli induced search-like eye movements to some extent in the after-task(stimulus) phase.

Another possibility is that preparation for the next trial would induce the tendency to fixate on the center of the display in the after-task phases. We presumed that participants looked at the center of the display to anticipate the appearance of the fixation point at the end of the trial because they were instructed to look at the fixation point when it appeared. Since participants naturally faced the display, we also presumed that the eyes would return to align with the head orientation after participants finished each trial when there was no specific visual search task to start searching efficiently in the next trial. However, our experimental design did not enable us to determine whether such eye movements are induced purely by the presentation of the visual stimuli; just the inertia of visual search in the preceding task displays, head orientation, or display center in the after-task phases in preparation for the next trial. One fact that favors the former possibility is that, as we discuss in detail in the next section, there was no effect of task difficulty of visual search on any eye movements in the after-task phases, indicating that inertia of visual search is unlikely.

In summary, when no task demand for visual search was eliminated in the after-task phases, there were less eye movements, i.e., saccade frequency and amplitude decreased and the variety of eye-movement patterns also decreased. In addition, eyes tended to fixate in the central region of the display in the after-task phase. A characteristic eye-movement pattern, a backward saccade, was also found in the after-task phase, but the impact of stimulus-driven control was limited.

### Aftereffects of task difficulty of preceding task on non-goal-driven eye movements

The RTs for manual responses to a target were faster in the easy task than in the difficult task. This shows that our manipulation of task difficulty by changing feature combinations between a target and a distractor was successful, and can produce different levels of cognitive workloads under these conditions.

The saccade amplitude and frequency both increased with task difficulty in the task phase (Figure 03). This result is similar to that reported by Zelinsky and Sheinberg (36), in which the saccade frequency was lower during parallel search, and to the result reported by Young and Hulleman (35), in which the saccade frequency increased with the task difficulty of a visual search task. We suggest that the saccade amplitude and frequency are affected by task difficulty because the difficult task required more saccades and larger amplitudes to efficiently find the target. More importantly, the effect of task difficulty on the saccade amplitude and frequency was not found in the after-task phases. This shows that eye movements in the after-task phases were independent of those in the task phase.

As shown in Figure 06, the gaze-shift pattern reflects task difficulty. The ratio of pattern 1 was much higher in the easy task than in the difficult-task. We assumed that the participants' eyes would be guided by the popped-up target as soon as the easy-task display was shown, and that the first saccade almost reached the target, after which the second saccade finished reaching the target. Thus, the ratio of pattern 1, which could be considered a corrective saccade, was much higher in the easy task than in the difficult task.

The ratios of patterns 3 and 4 were much higher in the difficult task than in the easy task. In the difficult task, the participants needed more of a scanning gaze-shift pattern to find the target. Thus, the ratios of patterns 3 and 4, which could be considered scanning saccades, were much higher in the difficult task than in the easy task.

Again, the gaze-shift pattern in the after-task phases were not affected by the task difficulty in the preceding visual search task, suggesting that eye movements in the after-task phases did not contain components of inertia of the preceding eye movements, or after effects due to the preceding cognitive workload.

### Gaze-shift-pattern model among phases and difficulties

**Figure 08.**
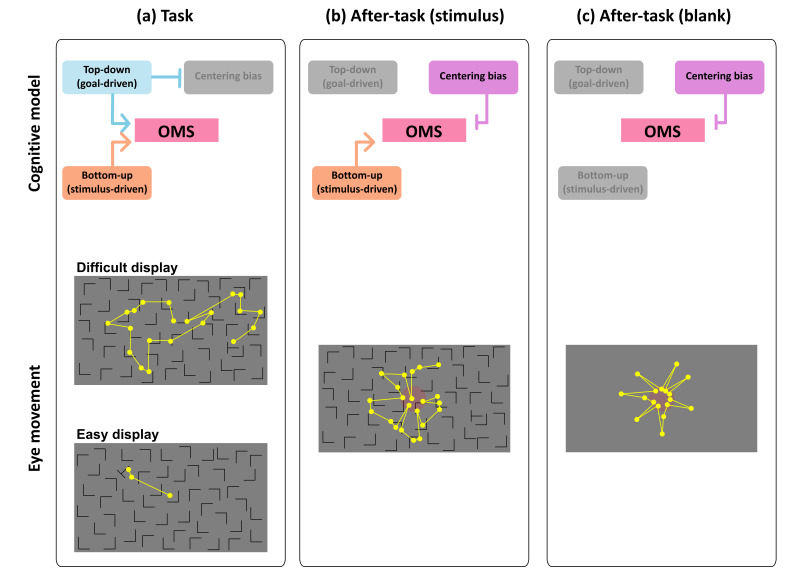
Cognitive model of OMS and eye movement patterns in all phases. Each after-task phase was conducted following task phase. Yellow dots represent fixation points, yellow lines represent saccades, and red circles represent center of display. These fixation points and saccades paths are examples.

We propose a conceptual model in the aftereffect of visual search (shown Figure 08) on the basis of the results of this study. We consider four factors affecting the OMS, which controls eye movements: top-down/goal-driven control, bottom-up/stimulus-driven control, centering bias, and on- or off-task states. Basically, in the task phase, consistent with most models of eye movement in visual search, we argue that the OMS is controlled in both top-down/goal-driven and bottom-up/stimulus-driven manner, and the engagement level of such control changes with task difficulty. The OMS was more strongly dominated by top-down/goal-driven control in the difficult task than in the easy task. Thus, scanning saccade patterns (patterns 3 and 4) were more strongly induced (Figure 06), and the saccade frequency and amplitude were larger in the difficult task than in the easy task (Figure 03). In the easy task, however, top-down/goal-driven control affected the OMS less than in the difficult task because the target was more easily detected before the top-down/goal-driven control fully engaged to the task. Thus, such stimulus-driven control of eye movements induced more frequent corrective saccades in the easy task. In the task phase, the centering bias less would engage the OMS because of the effect of visual search dominants on the OMS (Figure 05).

In the after-task phases, we consider that top-down/goal-driven control engaged the OMS much less than in the task phase because no visual search task was given to participants. Thus, eye-movement patterns changed relative to the task phase. Eye movements of patterns 2 and 3 decreased, and those of pattern 5 increased. Saccade amplitude also decreased in the after-task phases. Therefore, modes of eye movements drastically changed due to the elimination of top-down task demands. It is also important to recall that the effect of task difficulty of visual search in the task phase did not remain during the after-task phases. The reason we used task difficulty manipulation was to investigate the aftereffect of cognitive workload in the after-task phases on the OMS processing in these phases. However, no aftereffect was found, suggesting that the OMS in the after-task phases works independently of that in the task phase.

When the top-down task demand was eliminated, we also argue that much less effect of top-down/goal-driven control on the OMS resulted in the fixation remaining in the central part of the display (i.e., centering bias). This may be due to the centering bias enabling the OMS to rest or prepare for a ready state, meaning that the gaze remained in a convenient location to look at the entire screen. Furthermore, the centering bias may have entailed directly backward saccades (pattern 5) in the after-task phases. Although we could not find clear reason eyes moved when no task demand was given, there was a strong bias toward going back to the original position in consecutive saccades in the after-task phases.

The effect of bottom-up/stimulus-driven control was found in certain eye movements. By comparing eye movements in the after-task(stimulus) phase with those in the after-task(blank) phase, the saccade patterns that played a role in searching for a target (patterns 3 and 4) were induced. As a result, the gaze position was more widely distributed in the After-task(stimulus) phase than in the after-task(blank) phase (Figure 05). Eye movements were more frequent in the after-task(stimulus) phase than in the After-task(blank) phase. These results suggest that the mere presence of visual stimuli induces a scanning-like pattern, to some extent, even when no task demand is given.

## Limitation

There were two limitations in this study. One is that the effect of non-visual stimuli on gaze patterns given when the preceding visual task also consisted of a blank display was not clarified. Therefore, we discussed the gaze pattern of different levels of the top-down effect which was induced by the effect of task difficulty. The other limitation is that the effect of non-visual stimuli on gaze patterns was obtained in only one type of visual task. Therefore, we need to try other visual tasks.

## Ethics and Conflict of Interest

The authors declare that the contents of the article are in agreement with the ethics described in http://biblio.unibe.ch/portale/elibrary/BOP/jemr/ethics.html and Author Ayumi Takemoto was employed by the company OMRON Corporation. The remaining authors declare that the research was conducted in the absence of any commercial or financial relationships that could be construed as a potential conflict of interest.
